#  Linear variability of gait according to socioeconomic status in elderly

**Published:** 2016-06-30

**Authors:** Paul Medina González

**Affiliations:** Departamento de Kinesiología, Facultad de Ciencias de la Salud, Universidad Católica del Maule, Talca, Chile

**Keywords:** Gait, biomechanical phenomena, socioeconomic factors, allostasis, aging

## Abstract

**Aim:**

To evaluate the linear variability of comfortable gait according to socioeconomic status in community-dwelling elderly.

**Method:**

For this cross-sectional observational study 63 self- functioning elderly were categorized according to the socioeconomic level on medium-low (n= 33, age 69.0 ± 5.0 years) and medium-high (n= 30, age 71.0 ± 6.0 years). Each participant was asked to perform comfortable gait speed for 3 min on an 40 meters elliptical circuit, recording in video five strides which were transformed into frames, determining the minimum foot clearance, maximum foot clearance and stride length. The intra-group linear variability was calculated by the coefficient of variation in percent.

**Results:**

The trajectory parameters variability is not different according to socioeconomic status with a 30% (range= 15-55%) for the minimum foot clearance and 6% (range= 3-8%) in maximum foot clearance. Meanwhile, the stride length consistently was more variable in the medium-low socioeconomic status for the overall sample (*p*= 0.004), female (*p*= 0.041) and male gender (*p*= 0.007), with values near 4% ​​(range = 2.5-5.0%) in the medium-low and 2% (range = 1.5-3.5%) in the medium-high.

**Conclusions:**

The intra-group linear variability is consistently higher and within reference parameters for stride length during comfortable gait for elderly belonging to medium-low socioeconomic status. This might be indicative of greater complexity and consequent motor adaptability.

## Abstract (es)

**Objetivo:**

Evaluar la variabilidad lineal de marcha confortable según el nivel socioeconómico en adultos mayores de la comunidad.

**Métodos:**

Participaron en este estudio observacional y transversal 63 adultos mayores autovalentes, los cuales se categorizaron según el estatus socioeconómico en medio-bajo (n= 33; edad 69.0 ± 5.0 años) y medio-alto (n= 30; edad 71.0 ± 6.0 años). Se solicitó a cada participante realizar marcha natural durante 3 min en un circuito elíptico de 40 metros, registrándose en video cinco zancadas las que se transformaron a fotogramas, determinándose mediante su promedio, el mínimo despeje del pie, máximo despeje del pie y la longitud de zancada. La variabilidad lineal intra-grupo se calculó mediante el porcentaje del coeficiente de variación.

**Resultados:**

La variabilidad de los parámetros de trayectoria no es diferente según el nivel socioeconómico con un 30% (rango= 15-55%) para el mínimo despeje del pie y 6% (rango= 3-8%) en el máximo despeje del pie. Por su parte, la longitud de zancada presenta sistemáticamente mayor variabilidad en el nivel socioeconómico medio-bajo para la muestra general (*p*= 0.004), género femenino (*p*= 0.041) y masculino (*p*= 0.007), siendo sus valores cercanos al 4% (rango= 2.5-5.0%) en el nivel medio-bajo y 2% (rango= 1.5-3.5%) en el medio-alto.

**Conclusiones:**

La variabilidad lineal intra-grupo es sistemáticamente mayor y dentro de parámetros de referencia en la longitud de zancada durante marcha confortable para adultos mayores pertenecientes al nivel socioeconómico medio-bajo. Esto sería indicativo de mayor complejidad y consecuente adaptabilidad motora.

## Introduction

While aging is defined as a heterogeneous process of irreversible and natural changes 1, this entails a diminution of the physiological reserve, which would significantly explain the risk of functional deficit in various work capacities 2. However, the emergence of these consequences depends on the particular characteristics of each subject 3.

Movement by bipedal locomotion is considered a central element of the expression of functionality in the human being 4,5; therefore, its pertinent and sensitive characterization ensures to define appropriate prevention interventions in health. Notwithstanding the foregoing, most assessments of gait do not consider proven predictive and sensitivity factors that significantly affect it, such as intra-group and intra-subject kinematic variability 6 and the relationship with an irregular supporting surface 7. In this regard, a functional gait must ensure a skilled and efficient expression in different types of surfaces, as this is the usual ecological context for the performance of subjects in both urban and rural settings. It has been suggested that the main consequence of its dysfunction is falls, which are considered as the highest morbidity problem in this age group 8; however, its clinical estimate is developed through tests that focus on a measure based on the individual performance of a subject 9, leaving aside the relationship with the environment or the regularity of its trajectory. In this context, it has been said that gait efficiency is a complex process which could be associated with temporary fluctuation of parameters 10 and environmental characteristics 4.

Currently, the most significant environmental regulators for quality of life in humans are access to information and acquisition of goods 11, which are considered as the main dimensions in the development of instruments relevant to the measurement of socioeconomic status (SES) 12. It has been documented that the environment would play a key role in the variability and corresponding performance of human gait 13-15; regarding this, research conducted in elderly population (EP) have shown differences in walking speed according to SES 16, which could be an indicator or predictor of fragility and functional dependence 17. Given this background, the purpose of this research is to evaluate the linear variability (LV) of comfortable gait (CG) according to the SES in self-functioning EP in community.

## Material and Methods

### Participants

Observational-type and cross temporality research. 63 EP from 4 groups of the city of Talca, Chile participated; a non-probability convenience sample was used. Contact with the groups was carried out by a personal interview between the investigator and their formal representatives. Later, in February 2014, participants were recruited, being requested to attend measurements while wearing comfortable clothes and shoes, to be then evaluated in morning sessions (09:00-11:30 h) developed in the facilities of the Universidad Católica del Maule (UCM). The requested tests were completely performed. Before starting measurements, each of the participants signed an informed consent which was approved by the Scientific Ethics Committee of the UCM (follow-up report No. 2/2014).

Inclusion criteria were controlled by applying the test of Preventive Medicine of the Elderly (EMPAM, for its initials in Spanish) 18, verifying the following: age between 60-75 years, self-functioning condition according to the *Evaluación Funcional del Adulto Mayor, parte A* (EFAM-Chile; Functional Assessment of the Elder-Chile, part A) 18, cognitively normal (abbreviated Mini Mental State Examination ≥13 points) 18 and without established depression (Yessavage Scale <5 points) 18. The EFAM-Chile is a screening tool for the comprehensive functional assessment of EP, which was designed to predict the loss of physical, mental and social functioning. It sets diagnostic categories called self-functioning without risk, self-functioning with risk, and risk of dependency 19. This instrument has been validated by the behavior of indicators of fragility according to their diagnostic categories 20. 

Meanwhile, there were excluded subjects with uncompensated chronic diseases, established risk of falls (Tests "Unipodal Stance" and "Timed up and Go" positive) 18, moderate sequelae of neurological or cardiovascular diseases, and moderate lower limb pain (Analogous Visual Scale >3).

The SES was determined by applying the ADIMARK survey 12. The medium-low SES (ML) considered the C3 and D groups; while the medium-high SES (MH), the Abc1 and C2 categorization.

### Measurements

After measuring the functional (EFAM-Chile) and anthropometric status according to specific stratification of body mass index of the Chilean EP 18, was applied a photogrammetric protocol in accordance with a specific proposal documented 21. They were asked to walk naturally for 3 min on a 40 m elliptical circuit. In this regard, a camera was strategically located in the sagittal plane (Sony Handycam HDR-XR550) in an area called "registration", at a distance of 4 m to capture a video of each stride (5 strides in total) executed by the EP. Each record measurement was carried out posterior to the first 15 m path from the starting circuit area.

Subsequently, the video records were stored on a laptop computer (Toshiba^®^, model NB505-SP0115LL). The simple kinematic analysis was developed at a rate of 30 frames per second through a program of free access (TRACKER version 4.8 for Windows) 21. In order to monitor the recovery of the participants, the physiological variables heart rate and blood pressure were measured at the end of the test execution. 

### Determining variables for trajectory and distance

The operational definition of the path kinematic variables considers the minimum foot clearance (MFC) as the lowest height between the antero-inferior border of the foot and the ground 21, being obtained in the late rolling phase of gait 22. The maximum clearance of the foot (MaxFC) represents the largest height between the antero-inferior border of the foot and the ground 21, this value is determined during the early swing phase of gait 22. Meanwhile, the stride length (SL), is defined as the distance to make a complete gait cycle, which comprises the antero-lower vertex of the foot at the beginning and the end of a stride 21,23. The measurement unit used for all kinematic variables was the meter.

The calculation of the kinematic variables was performed by analyzing frames, considering a demarcation process that has shown a good reliability and applicability level 21. The procedure was developed by an external evaluator previously instructed in the protocol.

The percentage of LV for the kinematic parameters described was established by the following calculation formula: % CVkp = (Akp / SDkp) x 100; where, % CVkp is the percentage of the Coefficient of Variation of the kinematic parameter; Akp = Average of the 5 strides for the magnitude of the kinematic parameter; and SDkp = Standard Deviation of the 5 strides to the magnitude of the kinematic parameter. The formula was applied to every SES of the overall sample, female and male.

### Statistics

The contrast of normality was carried out with the Shapiro-Wilk test. The description of the variables was developed by average ± 1 standard deviation.

The LV of each kinematic parameter was established by the percentage of the coefficient of variation (% CV). The LV comparison, according to the SES and gender, was performed by the Mann-Whitney U test. The level of statistical significance was set at *p* ≤0.05. The statistical programs used were SPSS^®^, version 18.0; and GraphPad Prism^®^, version 5.0 (GraphPad Software Inc., San Diego, CA, USA).

##  Results 

From the point of view of general characteristics, participants in this research have an age range comprising the 65-75 years decade; and their nutritional status is mostly overweight (Table 1). Meanwhile, although the functional characterization presents a specific score superior for SES MH (*p *<0.001; Table 1), both operationalized SES are in the self-functioning ranking.

**Table 1.** Demographic and anthropometric characteristics of participants (N=63).

Socioeconomic status	Gender	n	Age (yrs)	Mass (Kg)	Height (m)	BMI (Kg/m2)	EFAM A (points)
Medium-low	F	25	69 ± 4	71.8 ± 9.8	1.52 ± 0.06	31.2 ± 4.3	49 ± 3
	M	8	68 ± 6	82.4 ± 12.7	1.64 ± 0.05	30.5 ± 3.1	51 ± 2
	Total	33	69 ± 5	74.1 ± 11.3	1.54 ± 0.07	31.0 ± 4.0	49 ± 3
Medium-high	F	23	70 ± 6	70.7 ± 13.0	1.53 ± 0.06	30.2 ± 4.7	52 ± 3
	M	7	74 ± 7	81.4 ± 10.6	1.69 ± 0.07	28.5 ± 4.2	52 ± 2
	Total	30	71 ± 6	72.9 ± 13.2	1.56 ± 0.09	29,8 ± 4.6	52 ± 3
p value			0.154	0.699	0.326	0.273	<0.001

Values are expressed as mean ± standard deviation for each variable.

F= Female; M = Male; n = number of participants per group; BMI = Body Mass Index; *EFAM A = Evaluación Funcional del Adulto Mayor parte A *(Functional Assessment of the Elder-Chile, part A). The *p value* established according to SES.

The intra-group LV for the analyzed trajectory indicators shows no statistically significant differences according to gender factors and SES (Table 2). In this regard, according to the behavior of 95% Confidence Intervals, the MFC presents a fluctuation between 14.9 and 57.1%; while in the MaxFC, the variability range is between 2.4 and 6.8%.

**Table 2.** Linear variability of comfortable gait according to socioeconomic status and gender.

Parameter	Gender	SES ML			SES MH			p value
		n	X ± DE	95% CI	n	X ± DE	95% CI	
Variability MFC†	F	25	26.7 ± 9.3	22.7-30.8	23	21.5 ± 9.2	17.5-25.4	0.056
	M	8	23.0 ± 10.6	15.6-30.9	7	36.0 ± 22.7	14.9-57.1	0.170
	Total	33	25.8 ± 9.6	22.4-29.2	30	24.9 ± 14.5	19.4-30.3	0.755
Variability MaxFC†	F	25	6.5 ± 2.4	5.6-7.6	23	5.6 ± 2.7	4.4-6.8	0.079
	M	8	5.9 ± 2.5	3.7-7.5	7	3.8 ± 1.5	2.4-5.2	0.249
	Total	33	6.3 ± 2.4	5.4-7.2	30	5.2 ± 2.5	4.2-6.1	0.074
Variability SL†	F	25	3.5 ± 1.6	2.9-4.2	23	2.6 ± 1.6	1.9-3.2	0.041
	M	8	3.3 ± 1.2	2.3-4.1	7	1.7 ± 0.7	1.1-2.3	0.007
	Total	33	3.5 ± 1.5	3.0-3.9	30	2.4 ± 1.5	1.8-2.9	0.004

† Percentage

It presents the mean ± standard deviation for the percentage of the Coefficient of Variation (%CV) for each kinematic variable in relation to registered 5 steps. F= Female; M = Male; n = number of participants per group.

For the analysis of linear variability comfortable gait according to socioeconomic status was used U Mann Whitney test. *p <0.05; **p <0.01

On the other hand, even though the behavior of the intra-group LV for SL is systematically higher in the SES ML for the overall sample (*p*= 0.004), female gender (*p*= 0.041) and male (*p*= 0.007), being the fluctuation registration obtained in most cases less than 5% for both SES analyzed.

## Discussion

The main finding of this exploratory research was that when performing gait, the SL behavior presents a higher LV in EP belonging to socioeconomic ML. In this scenario, the variability associated with the expression of movement turns out to be a very attractive field for disciplines that contribute to biomedical and human movement sciences 24-27. It has been reported that gait variability would be an indicator of ontogeny maturity 24, aging 25, morbidity associated to imbalance 26, and a translator of mechanical and physiological efficiency 27.

Regarding the behavior of the MFC as a kinematic indicator of gait, it is interesting to note that for the feminine gender, it can be seen a trend to greater variability in the SES ML (Table 2), which could strengthen the idea of ​​a gait with more complex features for this stratum 28, given the requirements of the environment to labor needs and walkway conditions, this statement is justified by the fact that the MFC performance in both social groups is very close to the normal value reported in the literature 29, which would provide the "acceptable minima" for this expression . Meanwhile, in men, the analysis is difficult because of the small number of subjects and the irregular behavior of the data.

The analysis during the early swing phase reports an intra-subject variability of MaxFC that is close to 6% in the SES ML, and 5% in the SES MH (Table 2). In this regard, reports with similar information are unknown. However, it is interesting to note that it is consistently higher the variability of this indicator in subjects of both genders belonging to SES ML. Whereas this indicator of variability behaves in a general reference set for the musculoskeletal system 30, this demonstration would result in behavior adaptability of motion to land slopes, which during this phase of the gait cycle is crucial to overcome the challenges imposed by the environment. Surely the inability to achieve statistical significance could be caused by the small number of strides analyzed and the number of subjects.

The stride length variability behavior reports statistically significant results by SES in both genders (Table 2). Moreover, it is observed that most of the data are under 6% for SES ML, and 3.5% for SES MH. In 1984, Gabell & Nayak analyzed the SL intra-group variability in healthy EP, reporting variation coefficient values that were less than 6% 31. Meanwhile, Beauchet *et al*. 2005, assessed in young subjects the behavior of stride variability in different spatiotemporal parameters, with and without the implementation of additional tasks (dual), reporting values ​​close to 4% in both experimental situations 32; the interesting thing is that the most affected expression would be the SL, which is less than 1,400 mm, being less than that reported in the literature 7. In this scenario, the analysis of intra-subject variability of spatial gait parameters has clinical relevance for the specific and early diagnosis of gait's function and dysfunction in EP.

The exclusive representation of movement through measures of central tendency is incomplete, because the normal motor expression does not represent a point in space or time, given its complexity to account for the challenges posed by the environment under the conceptual framework of adaptability 33; thus, it becomes necessary to determine normal ranges of a quality or quantity of specific movement. Therefore, if the adaptive motor range is outside these "acceptable limits," it would represent in subjects an important indicator of deficit or motor immaturity, resulting in movement dysfunctions.

**Figure 1.**
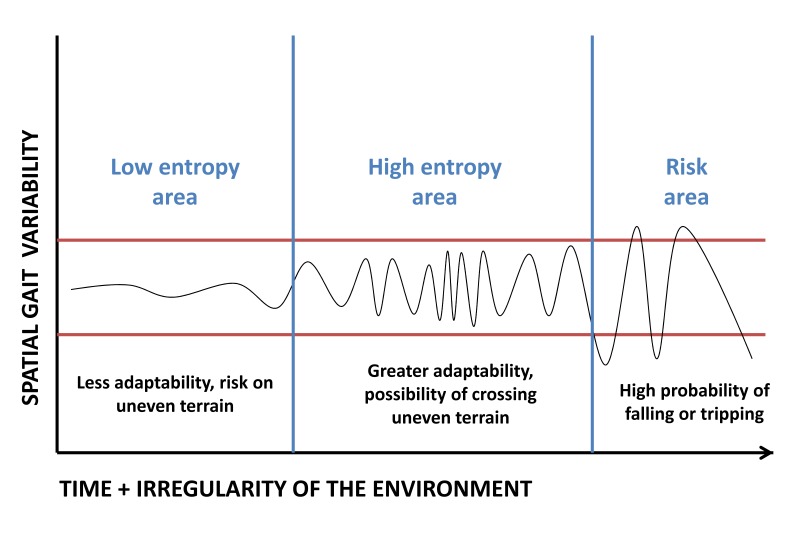
Scheme of gait variability associated with the environment. With the increasing irregularity of a given surface over time, the behavior of the variability of intra-subject gait is challenged to adapt to the environmental context (area of ​​high entropy); if this increase in variability is not achieved, it will not be possible to perform in more complex surfaces (area of ​​low entropy). On the other hand, if this response exceeds the limits of reference movement (horizontal lines), it translates risk for lack of motor control. It should be noted that this proposal would apply to any physiological and mechanical variable of gait.

It is known that socioeconomic factors influence morbidity and population functionality^34^-^36^, data that give support to the hypothesis of this research. However, human gait is complex and of non-linear character, since it is a process of multi-systemic physiological signals with irregular fluctuations 27. Thus, a high variability within normal limits represent a greater adaptive capacity; this situation is displayed in the present study, given the behavior of the SL (Table 2). It is interesting to recapitulate the entropy concept 37, which comes from thermodynamics, and that applies to the gait for quantify the regularity of a closed system, in this case within the boundaries of normality or reference 27,28 validated for a specific population. In this scenario and considering functionality as an indicator of the interaction between motor ability of living beings and the ecological environment 38, the aging process reduces the entropy within this system, triggering a movement that is less adaptable to environmental irregularities. Therefore, variability of gait within certain limits would be a signal of adaptability to movement, and also functional reserve for motor learning (Fig. 1). Thus, the allostatic mechanisms of biological systems guarantee to make changes within certain stability, unleashing with this physiological homeostasis, and in the case of gait, the acquisition of adaptability to contingencies of environmental and related requirements to the aging process.

Within the limitations of this research, it is the fact that gender groups are not comparable in number. Besides, from the point of view of external validity, the characteristics of subjects' recruitment do not allow an elaborated extrapolation of results, so it is necessary to contemplate this methodological strategy in future experiences. Similarly, it must be highlighted the high variability of the MFC; this situation could be explained because it is an indicator with a very small margin of expression, and kinematics catching is difficult given the high rate of registration, which is usually close to 4.6 m/s 39, which represents three times the speed of the center of body mass reported for CG in functional EP 17. In this regard, although the application of this technique of kinematic measurement in two planes has shown acceptable levels of reliability and outstanding levels of applicability 21, it is recommended for future research that consider the measurement of this path parameter, the use of cameras with a frequency of capture higher than the one used in the present study. 

From the standpoint of the projections, it is known that the magnitude of the variability does not change in healthy EP; however, gait dynamics changes with the aging process 40, so it is expected in future investigations to evaluate this behavior in various age ranges. Considering this conceptual stage, Costa *et al*., used the approximate entropy method to evaluate the progress at different speeds; they found out differences in all indicators 41. Thus, it looms the developing of new research models which integrate as analysis variables both gait speed and the measurement of temporal parameters 42. In this context, it is expected that this proposal will complement the already developed in the clinical field for guidance, upon determination of normal values ​​of the analyzed kinematic variables, diagnosis and therapeutic interventions to characterize and pertinently resolve dysfunctions of human movement.

Finally, when evaluating the results of this research, there is more LV for EP SL in the community belonging to the SES ML, which would be indicative of a more complex comfortable gait and consequent motor adaptability.

## References

[1] Weinert BT, Timiras PS (2003). Invited review: Theories of aging. J Appl Physiol.

[2] Chen X, Mao G, Leng SX (2014). Frailty syndrome: an overview. Clin Interv Aging.

[3] Durakovic Z, Misigoj-Durakovic M (2006). Does chronological age reduce working ability. Coll Antropol.

[4] Hutchinson JR, Gatesy SM (2001). Bipedalism.

[5] Jacelon CS (1986). The Barthel Index and other indices of functional ability. Rehabilitation Nursing.

[6] Jordan K, Challis JH, Newell KM (2007). Walking speed influences on gait cycle variability. Gait Posture.

[7] Merryweather A, Yoo B, Bloswick D (2011). Gait characteristics associated with trip-induced falls on level and sloped irregular surfaces. Minerals.

[8] Campbell AJ, Borrie MJ, Spears GF, Jackson SL, Brown JS, Fitzgerald JL (1990). Circumstances and consequences of falls experienced by a community population 70 years and over during a prospective study. Age Ageing.

[9] Perell KL, Nelson A, Goldman RL, Luther SL, Prieto-Lewis N, Rubenstein LZ (2001). Fall risk assessment measures: an analytic review. J Gerontol A Biol Sci Med Sci.

[10] Begg R, Best R, Dell'Oro L, Taylor S (2007). Minimum foot clearance during walking: strategies for the minimization of trip-related falls. Gait Posture.

[11] Rebato E, Susanne C, Chiarelli B (2005). Para comprender la antropología biológica.

[12] Adimark (2000). Manual de aplicación del nivel socioeconómico ESOMAR.

[13] Katsavelis D, Mukherjee M, Decker L, Stergiou N (2010). The effect of virtual reality on gait variability. Nonlinear Dynamics Psychol Life Sci.

[14] Hollman JH, Brey RH, Robb RA, Bang TJ, Kaufman KR (2006). Spatiotemporal gait deviations in a virtual reality environment. Gait Posture.

[15] Pickhinke J, Chien JH, Mukherjee M (2014). Varying the speed of perceived self-motion affects postural control during locomotion. Stud Health Technol Inform.

[16] Brunner E, Shipley M, Spencer V, Kivimaki M, Chandola T, Gimeno D (2009). Social inequality in walking speed in early old age in the Whitehall II study. J Gerontol A Biol Sci Med Sci.

[17] Fritz S, Lusardi M (2009). White paper: "walking speed: the sixth vital sign". J Geriatr Phys Ther.

[18] Ministerio de Salud Chile,Programa de Salud del Adulto Mayor, División de Prevención y Control de Enfermedades, Subsecretaría de Salud Pública Manual de Aplicación del Examen de Medicina Preventiva del Adulto Mayor (EMPAM).

[19] Ministerio de Salud (2008). Guía Clínica Examen de Medicina Preventiva.

[20] Tapia CP, Valdivia-Rojas Y, Varela HV, Carmona AG, Iturra VM, Jorquera MC (2015). Indicadores de fragilidad en adultos mayores del sistema público de salud de la ciudad de Antofagasta. Rev Med Chil.

[21] Medina P (2014). Confiabilidad de una metodología aplicable para la medición de cinemática simple del pie en adultos mayores autovalentes de la comunidad. Biosalud.

[22] Karst GM, Hageman PA, Jones TF, Bunner SH (1999). Reliability of foot trajectory measures within and between testing sessions. J Gerontol A Biol Sci Med Sci.

[23]  Muro-de-la-Herran A,  Garcia-Zapirain B,  Mendez-Zorrilla A (2014). Gait analysis methods: an overview of wearable and non-wearable systems, highlighting clinical applications. Sensors (Basel).

[24] Hausdorff JM, Zemany L, Peng CK, Golderger L (1999). Maturation of gait Dynamics: stride to stride variability and this temporal organization in children. J Appl Physiol (1985).

[25] Callisaya ML, Buzzard L, Smhmidt D, MCGinley JL, Srikanth K (2010). Ageing and gait variability - a population-based study of older people. Age Ageing.

[26] Khandoker AH, Palaniswami M, Begg RK (2008). A comparative study on approximate entropy measure and poincaré plot indexes of minimum foot clearance variability in the elderly during walking. J Neuroeng Rehabil.

[27] Hausdorff JM (2005). Gait variabilitymethods, modeling and meaning. J Neuroeng Rehabil.

[28]  De La Cruz TB ,  Sánchez LMD,  Sarabia CE,  Naranjo OJ (2013). Entropy in the analysis of gait complexity: A state of the art. British J Appl Sci Technol.

[29] Barrett RS, Mills PM, Begg RK (2010). A systematic review of the effect of ageing and falls history on minimum foot clearance characteristics during level walking. Gait Posture.

[30] Stokes M (1985). Reliability and Repeatability of methods of measuring muscle in physiotherapy. Physioth Pract.

[31] Gabell A, Nayak US (1984). The effect of age on variability in gait. J Gerontol.

[32] Beauchet O, Allali G, Annweiler C, Bridenbaugh S, Assal F, Kressig RW (2009). Gait variability among healthy adults: low and high stride-to-stride variability are both a reflection of gait stability. Gerontology.

[33] Allen DD (2007). Proposing 6 dimensions within the construct of movement in the Movement Continuum Theory. Phys Ther.

[34] Koster A, Penninx B, Bosma H, Kempen G, Harris T, Newman AB (2005). Is there a biomedical explanation for socioeconomic differences in incident mobility limitation. J Gerontol A Biol Sci Med Sci.

[35] Nilsson CJ, Avlund K, Lund R (2011). Onset of mobility limitations in old age: the combined effect of socioeconomic position and social relations. Age Ageing.

[36] Thorpe R, Koster A, Kritchevsky S, Newman AB, Harris T, Ayonayon HN (2011). Race, socioeconomic resources, and late-life mobility and decline: findings from the health, aging, and body composition study. J Gerontol A Biol Sci Med Sci.

[37] Pincus SM, Goldberger AL (1994). Physiological time-series analysis: what does regularity quantify?. Am J Physiol.

[38] Nathan R, Getz WM, Revilla E, Holyoak M, Kadmon R, Saltz D (2008). A movement ecology paradigm for unifying organismal movement research. Proc Natl Acad Sci U S A.

[39] Khandoker AH, Lynch K, Karmakar CK, Begg RK, Palaniswami M (2010). Toe clearance and velocity profiles of young and elderly during walking on sloped surfaces. J Neuroeng Rehabil.

[40] Karmakar CK, Khandoker AH, Begg RK, Palaniswami M, Taylor S Understanding ageing effects by approximate entropy analysis of gait variability.

[41] Costa M, Peng C-K, Goldberger AL, Hausdorff JM (2003). Multiscale entropy analysis of human gait dynamics. Physica A: Statist Mechanics Applicat.

[42] White DK, Neogi T, Nevitt MC, Peloquin CE, Zhu Y, Boudreau RM (2013). Trajectories of gait speed predicts mortality in well-functioning older adults: the Health, Aging and Body Composition study. J Gerontol A Biol Sci Med Sci.

